# HOMA-IR and the Matsuda Index as predictors of progression to type 1 diabetes in autoantibody-positive relatives

**DOI:** 10.1007/s00125-023-06034-y

**Published:** 2023-11-02

**Authors:** Alessandra Petrelli, Federica Cugnata, Debora Carnovale, Emanuele Bosi, Ingrid M. Libman, Lorenzo Piemonti, David Cuthbertson, Jay M. Sosenko

**Affiliations:** 1 Diabetes Research Institute, IRCCS Ospedale San Raffaele, Milan, Italy; 2 Vita-Salute San Raffaele University, Milan, Italy; 3 Division of Endocrinology, Diabetes and Metabolism, University of Pittsburgh and UPMC Children’s Hospital of Pittsburgh, Pittsburgh, PA USA; 4 Health Informatics Institute, University of South Florida, Tampa, FL USA; 5 Division of Endocrinology, Diabetes, and Metabolism, University of Miami, Miami, FL USA

**Keywords:** HOMA, Insulin resistance, Insulin sensitivity, Progression of type 1 diabetes, Stage 3, Type 1 diabetes

## Abstract

**Aim/hypothesis:**

We assessed whether HOMA-IR and the Matsuda Index are associated with transitions through stages of type 1 diabetes.

**Methods:**

Autoantibody (AAb)-positive relatives of individuals with type 1 diabetes (*n*=6256) from the TrialNet Pathway to Prevention were studied. Associations of indicators of insulin resistance (HOMA-IR) and insulin sensitivity (Matsuda Index) with BMI percentile (BMIp) and age were assessed with adjustments for measures of insulin secretion, Index60 and insulinogenic index (IGI). Cox regression was used to determine if tertiles of HOMA-IR and Matsuda Index predicted transitions from Not Staged (<2 AAbs) to Stage 1 (≥2 AAbs and normoglycaemia), from Stage 1 to Stage 2 (≥2 AAbs with dysglycaemia), and progression to Stage 3 (diabetes as defined by WHO/ADA criteria).

**Results:**

There were strong associations of HOMA-IR (positive) and Matsuda Index (inverse) with baseline age and BMIp (*p*<0.0001). After adjustments for Index60, transitioning from Stage 1 to Stage 2 was associated with higher HOMA-IR and lower Matsuda Index (HOMA-IR: HR=1.71, *p*<0.0001; Matsuda Index, HR=0.40, *p*<0.0001), as with progressing from Stages 1 or 2 to Stage 3 (HOMA-IR: HR=1.98, *p*<0.0001; Matsuda Index: HR=0.46, *p*<0.0001). Without adjustments, associations of progression to Stage 3 were inverse for HOMA-IR and positive for Matsuda Index, opposite in directionality with adjustments. When IGI was used in place of Index60, the findings were similar.

**Conclusions/interpretation:**

Progression to Stages 2 and 3 of type 1 diabetes increases with HOMA-IR and decreases with the Matsuda Index after adjustments for insulin secretion. Indicators of insulin secretion appear helpful for interpreting associations of progression to type 1 diabetes with HOMA-IR or the Matsuda Index in AAb-positive relatives.

**Graphical Abstract:**

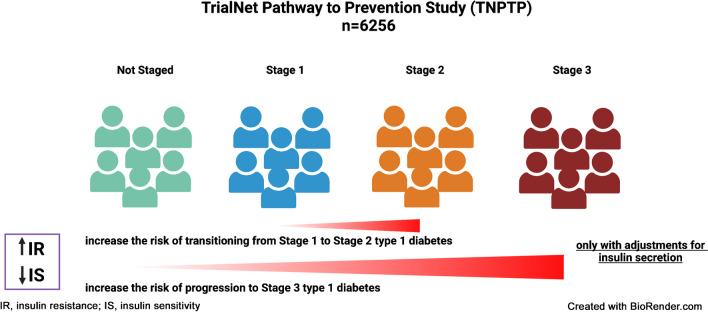

**Supplementary Information:**

The online version contains peer-reviewed but unedited supplementary material available at 10.1007/s00125-023-06034-y.


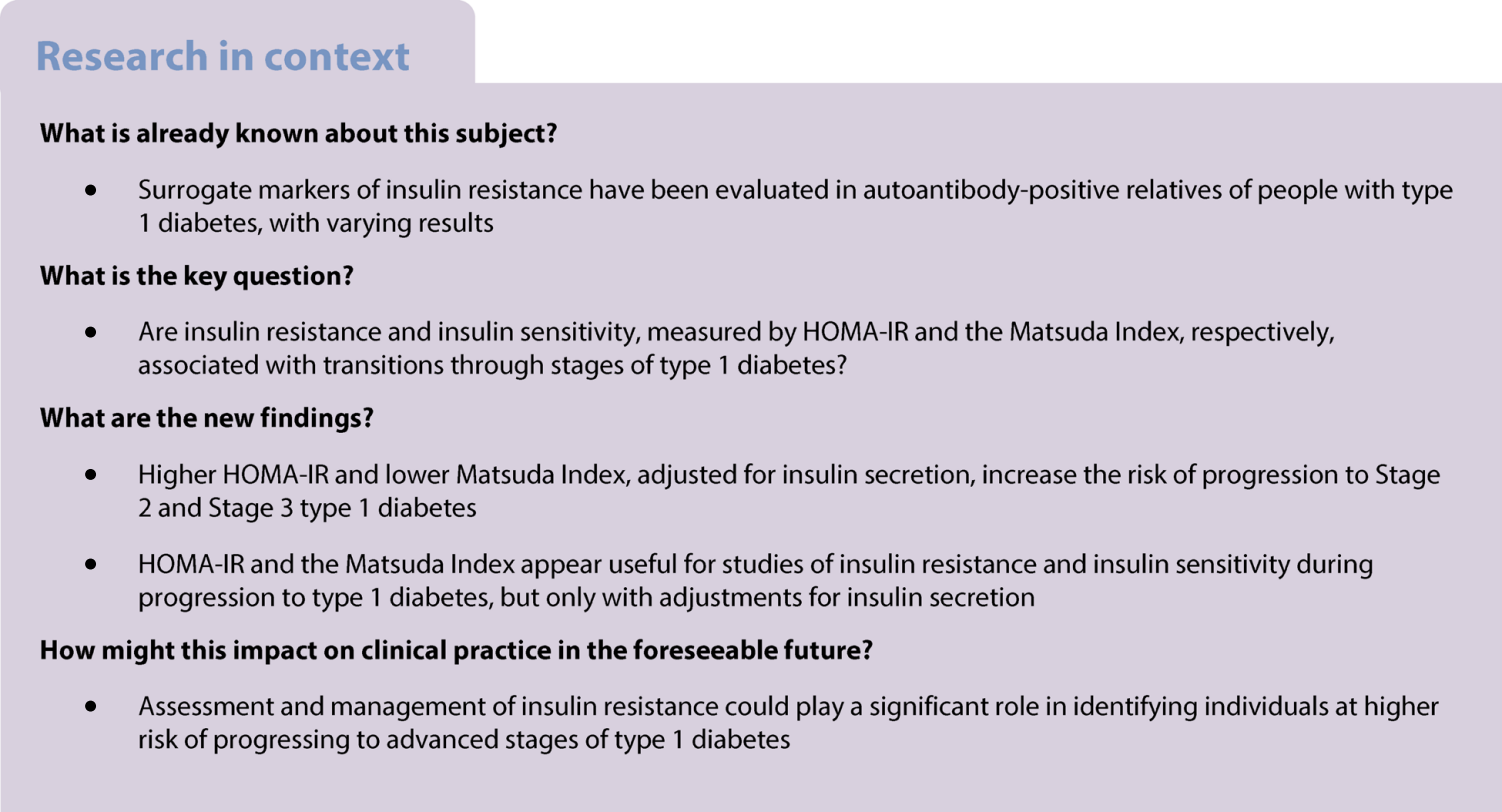


## Introduction

Diabetes is a heterogeneous group of metabolic disorders characterised by hyperglycaemia [1]. Based on the timing of disease onset, genetic predisposition and clinical phenotype, diabetes has been traditionally classified into two main groups: type 1 diabetes, featured by an autoimmune-mediated targeting of pancreatic beta cells leading to a deficiency in insulin secretion, and type 2 diabetes, tightly associated with obesity and ageing, featured by insulin resistance and chronic inflammation in insulin-sensitive tissues [2]. However, several phenotypic and mechanistic factors are shared between the two conditions, which has led to the hypothesis that they may in part share underlying mechanisms [3–6].

It is now accepted that a diagnosis of type 1 diabetes is usually preceded by a prolonged phase during which disease can be identified by measuring islet autoantibodies (AAbs). Pre-symptomatic stages of type 1 diabetes are defined by the presence of AAb ≥2 and normal glucose tolerance (Stage 1) or dysglycaemia (Stage 2), while individuals with <2 AAb and normal glucose tolerance, at lower risk of developing the disease [7], are considered ‘Not Staged’. The growing number of overweight/obese individuals with type 1 diabetes [8–10], together with evidence that excess body weight [11–14] increases the risk of developing type 1 diabetes, highlights the need to explore whether insulin resistance or insulin sensitivity are possible factors for type 1 diabetes development.

HOMA-IR and the Matsuda Index, validated in the general population, are commonly used indicators for assessments of insulin resistance and insulin sensitivity, respectively [15, 16]. However, using HOMA-IR and the Matsuda Index in AAb+ relatives may be problematic, since insulin and glucose levels, altered by deficient insulin secretion, are basic to their calculations. Surrogate measures of insulin resistance have been evaluated in AAb+ relatives with varying conclusions [11, 17], possibly because of the confounding effect of abnormal insulin secretion. Thus, adjustments for insulin secretion could be useful for the assessment of insulin resistance and sensitivity with HOMA-IR and the Matsuda Index in AAb+ relatives. The insulinogenic index (IGI) [(30 min insulin – fasting insulin)/(30 min glucose – fasting glucose)] has been utilised as a surrogate measure for insulin secretion in several studies [18–20]. Although Index60 has mainly been studied as a metabolic endpoint in AAb+ relatives [21, 22], by virtue of its constituents [log_*e*_ fasting C-peptide, 60 min glucose, 60 min C-peptide], it could also serve as an indicator of insulin secretion.

We assessed whether the IGI and Index60 can be used to adjust for HOMA-IR and Matsuda Index in examining the roles of insulin resistance and insulin sensitivity in the pathogenesis of type 1 diabetes. This was accomplished by evaluating the ability of HOMA-IR or Matsuda Index to predict stages of type 1 diabetes development, with and without adjustments for indicators of insulin secretion.

## Methods

### Characteristics of the study population

**Fig. 1:**
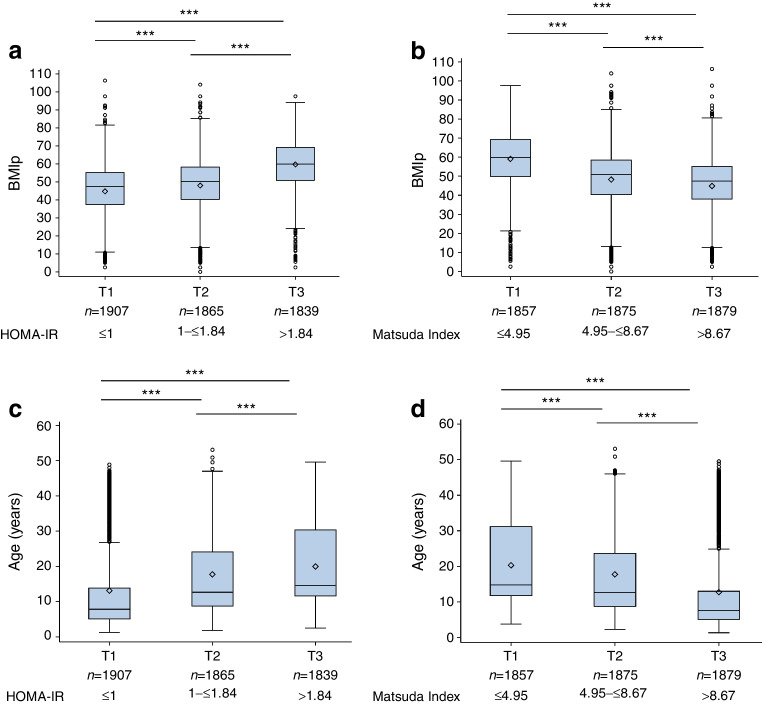
Box and whisker plots of BMIp and age stratified by tertiles based on the levels of HOMA-IR (**a**, **c**) and the Matsuda Index (**b**, **d**) among the study cohort of AAb+ relatives. Box and whisker plots display median, mean (diamond), first and third quartile, non-outlier minimum and maximum, and outliers. Number of individuals per group, cut-off values for HOMA-IR and Matsuda Index tertiles, and statistical significance based on comparison of tertiles from Wilcoxon two-sample tests are shown: ***p<0.001

First- and second-degree relatives of individuals with type 1 diabetes were enrolled into the TrialNet Pathway to Prevention Study (TNPTP) at the international clinical centres of the TrialNet network [23]. Institutional Review Board approval of the study was obtained at all participating sites, and written informed consent and assent, as applicable, were obtained. Gender was not considered in the study design. Sex was taken into account both in the calculation of BMI *z* score/percentiles, as well a covariate in the multivariate models. Sex was self-reported or reported by the participant’s parents. All participants were screened for islet autoantibodies to GAD (GADA), insulin (microinsulin antibody assay, mIAA), and IA-2 (IA-2A). If any of these were positive in screening, ZnT8A and ICA were also tested (ZnT8A testing was incorporated into the protocol in 2011). TNPTP methods for measuring islet autoantibodies have been previously described [24]. Participants identified as autoantibody positive, as well as a small subset of those autoantibody negative, were monitored with autoantibody testing, HbA_1c_ and an OGTT at 6- or 12-month intervals depending on estimated risk, as described in electronic supplementary material (ESM) Fig. 1. A total of *n*=6256 relatives were included in this study, *n*=4459 <18 years old and *n*=1797 ≥18 years old. The number who had baseline OGTTs in the TNPTP was 7233. Baseline is defined as the initial monitoring visit when the initial OGTT was performed. Outliers for both Index60 (<−3 and >3) and BMI (<12 or >50) were set to ‘missing’.

### 2 h OGTT

Participants underwent an OGTT (oral glucose dose 1.75 g/kg, maximum 75 g) after an overnight fast. C-peptide (nmol/l), glucose (mmol/l) and insulin (pmol/l) measurements were performed in the fasting state and then after oral glucose intake at 30, 60, 90 and 120 min.

### Staging of type 1 diabetes

‘Stage 0’ has been used as a term for individuals with AAbs who do not meet criteria for the type 1 diabetes stages. However, the definitions for Stage 0 have differed [25, 26]. To avoid confusion, we have used the term ‘Not Staged’ to indicate those who did not meet criteria for staging: normal glucose levels and <2 AAbs. Therefore, for the longitudinal analysis, participants were classified into stages of type 1 diabetes as follows [7]: Not Staged was defined as the presence of AAb <2 with normoglycaemia; Stage 1 was defined as the presence of AAb ≥2 with normoglycaemia; Stage 2 was defined as the presence of AAb ≥2 associated with dysglycaemia (impaired fasting blood glucose [5.6–6.9 mmol/l], and/or impaired glucose tolerance [7.8–11 mmol/l at 2 h], and/or glucose ≥11.1 mmol/l at 30, 60 or 90 min during OGTT); Stage 3 (i.e. diabetes as defined by WHO/ADA) occurs once hyperglycaemia develops (fasting blood glucose ≥7 mmol/l, and/or blood glucose ≥11.1 mmol/l at 120 min during OGTT) [27, 28]. Among the participants studied in the TNPTP, *n*=260 individuals transitioned from Not Staged to Stage 1, *n*=839 individuals transitioned from Stage 1 to Stage 2, and *n*=1189 progressed from baseline to Stage 3.

### BMI percentile calculation

BMI was calculated by dividing the participants’ weight in kilograms by the square of the participants height in meters. For participants less than 18 years of age, BMI percentile (BMIp) was determined based on sex and age-specific CDC growth charts (https://www.cdc.gov/growthcharts/Extended-BMI-Charts.html; accessed March 2023). For participants 18 years of age or older, BMI percentiles were determined based on the sex-specific BMI distributions from the 2015–2016 NHANES survey (https://wwwn.cdc.gov/nchs/nhanes/continuousnhanes/default.aspx?BeginYear=2015; accessed March 2023).

### Indexes of insulin resistance, insulin sensitivity and beta cell function

Estimation of insulin resistance with HOMA-IR and insulin sensitivity with the Matsuda Index were obtained from OGTT data. HOMA-IR was calculated as described previously [29]; the Matsuda Index is calculated as 10,000/√ [fasting glucose (mmol/l) × fasting insulin (pmol/l)] × [mean glucose (mmol/l) × mean insulin (pmol/l) during OGTT] [15]. Beta cell function was measured from OGTTs using Index60 [30], and the insulinogenic index (IGI) [31] [ratio of insulin (pmol/l) at 30 min – fasting insulin (pmol/l) to glucose (mmol/l) at 30 min − fasting glucose (mmol/l)]. Negative values of IGI were not included in the analysis.

### Statistical methods

The Spearman correlation coefficient was calculated to evaluate the relationship between HOMA-IR and Matsuda Index. Linear regression models were utilised to assess the relationship of BMIp and age with HOMA-IR and Matsuda Index, both adjusted by Index60 and IGI independently. Tertiles of HOMA-IR and Matsuda Index were calculated. BMIp and age were compared by the tertiles of HOMA-IR and Matsuda Index, both overall using the Kruskal–Wallis test, and pairwise using the Wilcoxon two-sample test. Utilising a Bonferroni adjustment due to multiple tests, pairwise tests with a *p*<0.0167 were considered significant. The time from Not Staged to Stage 1, and the time from Stage 1 to Stage 2, were fit for HOMA-IR both unadjusted and adjusted for age and Index60 or IGI using Cox proportional hazard models. These models were repeated replacing HOMA-IR with Matsuda Index. Time from Not Staged to Stage 1, from Stage 1 to Stage 2 and from study entry to type 1 diabetes were compared by tertiles of HOMA-IR and Matsuda Index using Kaplan–Meir curves and the Logrank test. Additionally, Cox proportional hazard models for time to type 1 diabetes from study entry were fit for HOMA-IR both unadjusted and adjusted for age and Index60 or IGI. Again, these models were repeated replacing HOMA-IR with Matsuda Index. Participants who withdrew from the study, became lost to follow-up, or had an event that prevented them from experiencing the outcomes of interest (i.e. a competing risk such as death) were censored as of the last visit prior to the event. No method of imputation was utilised for missing data; participants who were missing parameters utilised in a particular model were excluded. Statistical analyses were performed with SAS v9.4 (Cary, NC, USA).

## Results

### Associations of HOMA-IR and the Matsuda Index with BMIp and age

Among the 6256 relatives of participants with type 1 diabetes enrolled in the TNPTP, most were children (*n*=4459) with a median age of 12.2 years. The following individuals were excluded from the study: 536 people with OGTT in the diabetic range and 441 people with missing insulin values. Out of the total 2650 Not Staged individuals, the majority (93.2%) had one autoantibody (data not shown). Across the study cohort there were 57.6% who had two or more autoantibodies and 23.4% with dysglycaemia. The median BMIp was 52.2. Median values of HOMA-IR and Matsuda Index were 1.36 and 6.56, respectively (Table 1).

**Table 1:** Characteristics of the study cohort

Characteristic	All participants *N*=6256
Age, median [IQR]	12.21 [7.87–21.28]
Male sex, *n* (%)	3038 (48.6)
Number of AAbs, *n* (%)	
0	180 (2.9)
1	2470 (39.5)
2	1451 (23.2)
3	963 (15.4)
4	741 (11.8)
5	451 (7.2)
Type of AAb, *n* (%)	
GAD65+	4955 (79.2)
IA-2+	1900 (30.4)
mIAA+	2524 (40.3)
ICA+	2217 (35.4)
ZNT8+	1635 (26.1)
AAb ≥2, *n* (%)	3606 (57.6)
HLA–DR3 and/or DR4^a^, *n* (%)	4543 (79.1)
Dysglycaemia, *n* (%)	1462 (23.4)
BMI, median [IQR]	19.47 [16.28–24.49]
Adults aged ≥18, median [IQR], *n*	26.09 [23.02–30.77], 1590
Children <18, median [IQR], *n*	17.39 [15.69–20.61], 4024
BMIp, median [IQR]	52.18 [42.24–61.07]
Adults aged ≥18, median [IQR], *n*	42.61 [23.32–65.50], 1589
Children <18, median [IQR], *n*	53.26 [46.03–60.47], 4022
Age of children <18 with a BMIp, median [IQR], *n*	9.62 [6.58–12.82], 4022
HOMA-IR, median [IQR]	1.36 [0.84–2.18]
Matsuda Index, median [IQR]	6.56 [4.23–10.26]
Index60, median [IQR]	0.04 [−0.69, 0.71]
IGI, median [IQR]	0.75 [0.41–1.31]

^a^HLA typing information was unavailable for 514 individuals

Consistent with evidence from populations validated for HOMA-IR and Matsuda Index [32, 33], their relationship was found to be curvilinear and inverse (ESM Fig. 2), with a Spearman correlation coefficient of −0.926 (*p*<0.001). When the study cohort was stratified into tertiles 1 to 3 (T1, T2 and T3) of HOMA-IR (Fig. 1a,c) or Matsuda Index (Fig. 1b,d), a positive association with HOMA-IR (*p*<0.001 for T1 vs T3) and an inverse association with Matsuda Index (*p*<0.001 for T1 vs T3) was found with both BMIp (Fig. 1a,b) and age (Fig. 1c,d).

Linear regression models were used to further assess the associations. There were significant positive associations of HOMA-IR with BMIp and age, and significant inverse associations of Matsuda Index with BMIp and age, with or without adjustments for Index60 (*p*<0.0001) (ESM Table 1) or IGI (*p*<0.0001) (ESM Table 2).

### Transition from Not Staged to Stage 1 according to HOMA-IR and Matsuda Index values

We performed a longitudinal analysis to determine whether insulin resistance or insulin sensitivity had a role in the transitioning between pre-symptomatic stages of type 1 diabetes development. Both HOMA-IR and Matsuda Index were divided into tertiles and then, using proportional hazards models, assessed whether there were associations of progression from Not Staged to Stage 1 according to those tertiles (Table 2). We found no significant differences for transitioning between T1 and T2, or between T1 and T3 of HOMA-IR or Matsuda Index, with and without adjustment for Index60 (Table 2) or IGI (ESM Table 3).

**Table 2:** Tertiles of HOMA-IR and Matsuda Index predict the risk of transitioning from Stage 1 to Stage 2, but not from Not Staged to Stage 1 type 1 diabetes

	HOMA-IR			Matsuda Index		
Parameter	Parameter estimate	*p* value	HR	Parameter estimate	*p* value	HR
Not Staged to Stage 1 (*n*=2399)						
T2 vs T1	−0.031	0.8359	0.969	0.214	0.1753	1.239
T3 vs T1	−0.076	0.6203	0.926	0.229	0.1446	1.258
^a^T2 vs T1	−0.013	0.9305	0.987	0.209	0.2135	1.233
^a^T3 vs T1	−0.025	0.8818	0.975	0.222	0.2221	1.249
Index60 (NS)	0.047	0.4882	1.049	0.005	0.9393	1.005
Stage 1 to 2 (*n*=3178)						
T2 vs T1	−0.131	0.1156	0.877	−0.288	0.0010	0.749
T3 vs T1	0.148	0.0768	1.160	−0.311	0.0002	0.732
^a^T2 vs T1	0.031	0.7128	1.032	−0.659	<0.0001	0.517
^a^T3 vs T1	0.534	<0.0001	1.707	−0.918	<0.0001	0.399
Index60 (St1)	0.385	<0.0001	1.470	0.498	<0.0001	1.647

Cox regression analysis (NS, Not Staged; St1, Stage 1); Not Staged (AAb <2 with normoglycaemia) to Stage 1 (AAb ≥2 with normoglycaemia): Index60 values at Not Staged used for adjustment; Stage 1 to Stage 2 (AAb ≥2 associated with dysglycaemia): Index60 values at Stage 1 used for adjustment

^a^Indented rows under Parameter heading indicate adjustment for Index60

### Transition from Stage 1 to Stage 2 according to HOMA-IR and Matsuda Index values

In analyses assessing a possible influence of HOMA-IR or Matsuda Index on progression from Stage 1 to Stage 2 (Table 2), there were no significant differences among HOMA-IR tertiles for transitioning from Stage 1 to Stage 2 without adjustments. However, T3 transitioned significantly more than T1 with an adjustment for either Index60 (HR for T3 vs T1 1.71 [95% CI 1.42, 2.05], *p*<0.0001; Table 2) or IGI (*p*=0.001; ESM Table 3).

T1 of Matsuda Index transitioned from Stage 1 to Stage 2 more than either T2 or T3 without adjustment for Index60 (HR for T2 vs T1 0.75 [0.63, 0.89], *p*=0.001; HR for T3 vs T1 0.73 [0.62, 0.87], *p*<0.001). Those differences were greater with adjustments for Index60 (Table 2) or IGI (*p*<0.0001 for all associations, ESM Table 3).

### Prediction of transition from Not Staged to Stage 1 and from Stage 1 to 2 according to HOMA-IR and Matsuda Index values

**Fig. 2:**
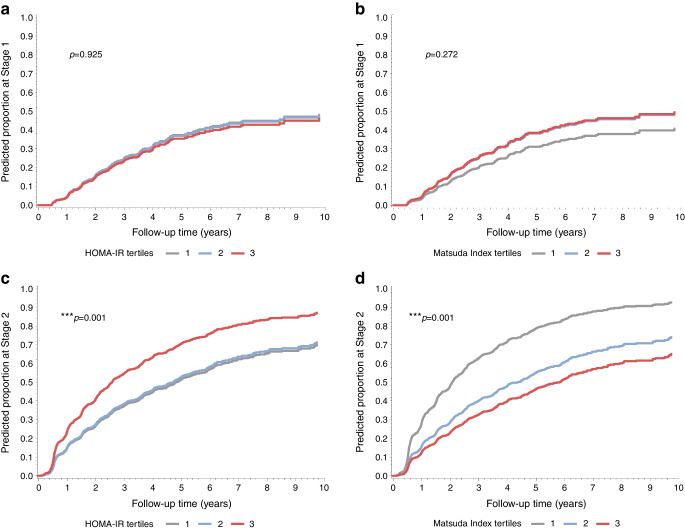
Predicted survival functions of time from Not Staged to Stage 1 (**a**, **b**) and from Stage 1 to Stage 2 (**c**, **d**) by tertiles of HOMA-IR (**a**, **c**) and the Matsuda Index (**b**, **d**) based on a Cox regression analysis adjusted for Index60. Estimates were determined based on the median value of Index60 (i.e. 0.04). ***p<0.001

Survival curves were constructed for the progression from Not Staged to Stage 1, and from Stage 1 to Stage 2 according to tertiles of HOMA-IR or Matsuda Index. The cumulative incidence from Not Staged to Stage 1 did not differ significantly between HOMA-IR tertiles (overall differences between HOMA-IR tertiles adjusted for Index60 [*p*=0.93; Fig. 2a] or adjusted for IGI [*p*=0.90; ESM Fig. 3a]), nor did they differ for Matsuda Index tertiles (overall differences between Matsuda Index tertiles adjusted for Index60 [*p*=0.27; Fig. 2b] or IGI [*p*=0.19]; ESM Fig. 3b).

In contrast, from Stage 1 to Stage 2 the cumulative incidence increased with higher HOMA-IR levels (overall differences between HOMA-IR tertiles adjusted for Index60 [*p*<0.001, Fig. 2c]; adjusted for IGI [*p*=0.0003, ESM Fig. 3c]), and decreased with lower Matsuda Index levels (overall differences between Matsuda Index tertiles adjusted for Index60 [*p*<0.001, Fig. 2d]; adjusted for IGI [*p*<0.0001, ESM Fig. 3d]). The cumulative incidence for T3 of HOMA-IR was significantly greater (*p*<0.0001) than the cumulative incidence of T1 and T2, whereas the cumulative incidence differed significantly between all tertile pairs of Matsuda Index (*p*<0.0001).

### Impact of age on transition through Stages of type 1 diabetes

We also examined the impact of age on the transition from Not Staged to Stage 1, and Stage 1 to Stage 2, and found that age was a strong inverse predictor of progression from Not Staged to Stage 1 (*p*<0.001 for all associations), but not from Stage 1 to Stage 2. Age did not influence the associations of progression with HOMA-IR or Matsuda Index (ESM Table 4).

### Prediction of Stage 3 according to HOMA-IR and Matsuda Index values

We assessed the risk of progression to Stage 3 (diagnosis of type 1 diabetes) based on tertiles of HOMA-IR or Matsuda Index at study entry (Table 3). ‘Study entry’ is defined as the monitoring visit at which the initial OGTT was performed. T2 and T3 of HOMA-IR had lower risks for Stage 3 than T1 (HR for T2 vs T1 0.77 [0.68, 0.88], *p*=0.0002; HR for T3 vs T1 0.67 [0.58, 0.77], *p*<0.0001), whereas T2 and T3 of Matsuda Index at baseline had higher risks than T1 (HR for T2 vs T1 1.19 [1.02, 1.38], *p*=0.02; HR for T3 vs T1 1.52 [1.31, 1.75], *p*<0.0001; Table 3). However, after adjusting for Index60, we found that, although the associations remained significant (*p*<0.001 for all), the directions were reversed: for HOMA-IR, T2 and T3 went from lower risk to higher risk than T1; for Matsuda Index, T2 and T3 went from higher risk to lower risk than T1 (HR for T3 vs T1 of HOMA-IR 1.98 [1.68, 2.27], *p*<0.0001; HR for T3 vs T1 of Matsuda Index 0.46 [0.40, 0.54], *p*<0.0001). Results were similar after adjustment for IGI (ESM Table 5). Associations of progression to Stage 3 with HOMA-IR and Matsuda Index tertiles remained significant when age was added as a covariate together with Index60 (Table 3) and with IGI (ESM Table 5).

**Table 3:** Tertiles of HOMA-IR and Matsuda Index at study entry predict the risk of developing Stage 3 type 1 diabetes

	HOMA-IR *n*=6114			Matsuda Index *n*=6256		
Parameter	Parameter estimate	*p* value	HR	Parameter estimate	*p* value	HR
T2 vs T1	−0.256	0.0002	0.774	0.173	0.0249	1.190
T3 vs T1	−0.406	<0.0001	0.666	0.415	<0.0001	1.516
^a^T2 vs T1	0.270	0.0001	1.311	−0.538	<0.0001	0.583
^a^T3 vs T1	0.681	<0.0001	1.977	−0.783	<0.0001	0.457
Index60 (baseline)	1.196	<0.0001	3.307	1.208	<0.0001	3.349
^b^T2 vs T1	0.254	0.0002	1.289	−0.618	<0.0001	0.539
^b^T3 vs T1	0.670	<0.0001	1.954	−0.952	<0.0001	0.386
Index60 (baseline)	1.193	<0.0001	3.298	1.157	<0.0001	3.183
Age (baseline)	−0.022	<0.0001	0.978	−0.024	<0.0001	0.976

Cox regression analysis with baseline Index60, or Index60 and age, as covariates for adjustment

^a^Indented rows under Parameter heading indicate adjustment for Index60

^b^Indented rows under Parameter heading indicate adjustment for Index60 and age

Baseline: monitoring visit when the initial OGTT was performed

**Fig. 3:**
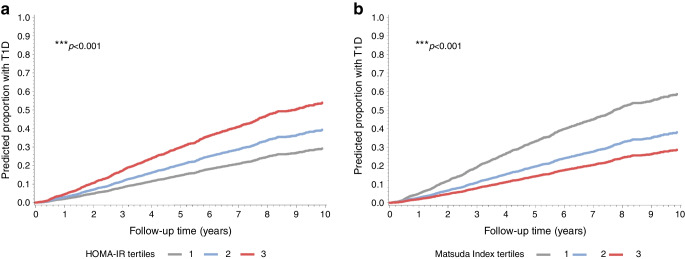
Predicted survival functions of time to type 1 diabetes by tertiles of HOMA-IR (**a**) and the Matsuda Index (**b**) based on a Cox Regression analysis adjusted for Index60. Estimates were determined based on the median value of Index60 (i.e. 0.04). ***p<0.001. T1D, type 1 diabetes

The cumulative incidence of type 1 diabetes over a 10 year period increased with higher levels of HOMA-IR (Fig. 3a) and increased with lower levels of Matsuda Index (Fig. 3b) after adjustments for Index60 (Fig. 3a,b) and IGI (ESM Fig. 4).

## Discussion

By analysing data from a cohort of 6256 AAb+ relatives of individuals with type 1 diabetes, we found that transitioning from Stage 1 to Stage 2 and progression to Stage 3 of type 1 diabetes was associated with increased levels of HOMA-IR and reduced levels of Matsuda Index, only when adjusted for insulin secretion.

Increased insulin resistance is a condition typically observed in individuals who have type 2 diabetes, with a role historically considered marginal among those with type 1 diabetes. However, in studies using the hyperinsulinaemic–euglycaemic clamp, the gold standard for assessing insulin action in vivo [34], insulin resistance is evident in participants with type 1 diabetes, involving both central and peripheral tissues [35–39]. Growing literature supports the hypothesis that insulin resistance is a condition that precedes symptomatic type 1 diabetes and contributes to the pathogenesis of the disease. Excess body weight in pre-symptomatic children was shown to be associated with a 63% increase in risk of developing type 1 diabetes [40]. Higher cumulative excess BMI conferred significantly greater risk of progressing to symptomatic disease [11]; this was also seen in overweight and obese adolescents [13]. A 10% increment in weight was associated with a 50–60% increase in risk of type 1 diabetes before the age of 3 years, while obesity after 3 years of age was associated with a twofold risk of developing the disease [14]. More recently, Galderisi et al [41] reported that Stage 1 in youth is associated with reduced insulin sensitivity, lower beta cell responsiveness and the presence of blunted insulin clearance, which highlights a possible role for insulin resistance and insulin sensitivity in the early stages of the natural history of type 1 diabetes.

Findings from this study suggest that insulin resistance and insulin sensitivity can be assessed using HOMA-IR and the Matsuda Index, respectively, in AAb+ relatives. However, importantly, adjustments with indicators of insulin secretion such as Index60 and IGI appear necessary. In fact, with these adjustments, the associations of transitioning from baseline to Stage 3 type 1 diabetes were reversed: the association with HOMA-IR went from inverse to positive, while the association with Matsuda Index went from positive to inverse. This is in line with data reported by Fourlanos et al [40] showing, in a small cohort of relatives positive for islet AAbs, that individuals who progressed to diabetes had a greater insulin resistance for their level of insulin secretion. Our data also align with previous studies that used IVGTT to measure insulin resistance or insulin sensitivity. These studies followed children or young adults who were relatives of people with type 1 diabetes, who had high genetic risk [42] or had two or more AAbs [43] to observe the development of type 1 diabetes. Both studies indicated that insulin resistance, measured by HOMA-IR, was an independent determinant of progression when adjusted for beta cell function, as measured by first-phase insulin release (FPIR). These data were further supported by a study of identical twins, which showed that an increased HOMA-IR relative to FPIR levels in AAb+ twins was associated with progression to type 1 diabetes [44]. This evidence is in line with the accelerator hypothesis [45], which indicates that increasing insulin resistance and reduced insulin sensitivity accelerate the disease process leading to type 1 diabetes only when whole glucose metabolism (secretion and peripheral action) is considered. Several studies have shown that, among relatives of individuals with type 1 diabetes, predictors of progression to diabetes include high-risk HLA genotypes, age at autoantibody seroconversion, increasing numbers of positive AAbs, and dysfunctional glucose-stimulated insulin secretion [46, 47]. Our data indicate that insulin resistance and insulin sensitivity are risk factors for the progression to type 1 diabetes. However, this study does not determine the relative significance of insulin resistance/sensitivity compared with the other factors.

Index60 was somewhat more impactful for associations of progression with HOMA-IR or Matsuda Index than IGI. Although both are composite measures of glucose and C-peptide, Index60 is based upon responsiveness at 60 min, whereas IGI is based on responsiveness at 30 min. If the influence of HOMA-IR and Matsuda Index on progression relates more to later insulin responsiveness, Index60 could be more relevant. However, the risk of type 1 diabetes was shown to increase with alterations in both early and late C-peptide secretion, with lower early C-peptide responses (30–0 min) and higher late C-peptide responses (120–60 min) being associated with increased risk of progression [48].

Based on the seminal role of glucose and insulin in calculating HOMA-IR and the Matsuda Index, and their inverse position in the HOMA-IR and Matsuda Index formulas, it should not be surprising that strengths of associations with transition through stages of type 1 diabetes tended to be similar and in opposite directions. The Matsuda Index, however, appears more definitive than HOMA-IR in predicting transition from Stage 1 to Stage 2. This is likely explained by HOMA-IR being a function of the fasting state, whereas the Matsuda Index is an indicator of whole-body insulin sensitivity and indicates how efficiently the body handles glucose after an oral glucose load [15]. Thus, the Matsuda Index would appear to have more capability for detecting deficiencies in the response to insulin action.

Prediction by HOMA-IR and the Matsuda Index for the progression from Stage 1 to Stage 2, but not from Not Staged to Stage 1, suggests a greater impact of insulin sensitivity and insulin resistance when progression to diabetes is more advanced. Yet, values of insulin resistance and insulin sensitivity at study entry were already predictive of the progression to diagnosis. This is in line with previously published literature assessing the effect of elevated BMI, an indicator for high insulin resistance and low insulin sensitivity, in relatives of individuals with type 1 diabetes [11, 40, 41]. Although our results show associations between the transition through stages of type 1 diabetes and measures of insulin resistance and insulin sensitivity, causality could not be determined. However, the consistency of the findings between the progression and the prediction of type 1 diabetes using HOMA-IR and the Matsuda Index suggest that increased autoimmunity and increased insulin resistance occur concomitantly. It would be worth investigating the possibility that autoimmunity and insulin resistance/sensitivity share the same pathogenic basis, as previously hypothesised by our group and by others [5, 6, 49]. A possible explanation of how decreased insulin secretion might cause metabolic changes leading to reduction of insulin sensitivity could involve the neuroendocrine system. Indeed, insulin deficiency results in a decrease in liver GH receptor (GHR) expression, increased visceral adiposity with elevated levels of circulating NEFA, and consequent inhibition of insulin receptor substrate-1 (IRS-1) activity [50].

A limitation of this study is that participants were retrospectively selected from among participants in the TNPTP observational study based on specific criteria (e.g. presence of an OGTT at the study visit) with a potential for bias. HOMA-IR and Matsuda Index measurements used in this analysis have not been validated for AAb+ populations. Thus, they should not be construed as being definitive measures of insulin resistance and insulin sensitivity in our study. Still, although deficient insulin secretion was a major impediment for applying HOMA-IR and Matsuda Index to our study population, the findings suggest that adjustments for insulin secretion can potentially provide insights into possible roles for insulin resistance and insulin sensitivity during the development of type 1 diabetes. The oral disposition index [51, 52] has provided evidence of the critical role of insulin secretion for assessing insulin resistance and insulin sensitivity in validated populations. Furthermore, evidence for the specific tissue sources of insulin resistance have previously been reported, with HOMA-IR reflecting hepatic insulin resistance and Matsuda Index reflecting whole-body insulin sensitivity [41]; however, there is not full agreement [42]. In this study, we are not able to address such specificity in our insulin-deficient population.

In conclusion, this study used the novel approach of adjusting for insulin secretion in assessing whether insulin resistance and insulin sensitivity, indicated by HOMA-IR and the Matsuda Index, respectively, are factors involved in the progression towards type 1 diabetes. With adjustments, progression from Stage 1 to Stage 2 was positively related to insulin resistance, and inversely related to insulin sensitivity. The prediction of type 1 diabetes risk was consistent with the staging findings: after adjusting for insulin secretion, type 1 diabetes occurrence was associated with high insulin resistance and low insulin sensitivity. As sex was taken into account in the modelling, we expect that the findings can be generalised to all sexes/genders. These results highlight the possible importance of targeting insulin resistance to delay the progression towards advanced stages of type 1 diabetes. They thus provide a rationale and a means for investigating therapeutic strategies, such as diet, metformin and/or glucagon-like peptide-1 (GLP-1) agonists, in combination with the targeting of autoimmunity, for preventing the progression to type 1 diabetes.

### Supplementary Information

Below is the link to the electronic supplementary material.

Supplementary file1 (PDF 389 KB)

## Data Availability

The data sets analysed for the present study are available from the corresponding author upon reasonable request.

## Abbreviations

AAb: Autoantibody
BMIp: BMI percentile
FPIR: First-phase insulin release
IGI: Insulinogenic index
T1, T2, T3: Tertile 1, 2, 3
TNPTP: TrialNet Pathway to Prevention Study
