# Supplementary material for: HOMA-IR and the Matsuda Index as predictors of progression to type 1 diabetes in autoantibody-positive relatives
Source: Diabetologia. 2023 Nov 2;67(2):290–300. doi: 10.1007/s00125-023-06034-y (PMC10789859; doi:10.1007/s00125-023-06034-y)
Supplement: Supplementary file 1 — Supplementary file1 (PDF 389 KB) [file 125_2023_6034_MOESM1_ESM.pdf]

Electronic supplementary material (ESM)

TABLES

	**HOMA-IR** N=5611				**MI** N=5611			
Variable	Parameter Estimate	P value	95% Confidence Limits		Parameter Estimate	P value	95% Confidence Limits	
Intercept	-0.498	<.0001	-0.553	-0.443	2.606	<.0001	2.555	2.656
BMIp	0.015	<.0001	0.014	0.016	-0.014	<.0001	-0.015	-0.013
Intercept	-0.389	<.0001	-0.439	-0.340	2.492	<.0001	2.449	2.535
BMIp	0.013	<.0001	0.012	0.014	-0.011	<.0001	-0.012	-0.011
Index60	-0.283	<.0001	-0.299	-0.268	0.294	<.0001	0.281	0.307
Intercept	0.048	0.0012	0.019	0.078	2.112	<.0001	2.085	2.139
Age	0.014	<.0001	0.013	0.015	-0.014	<.0001	-0.015	-0.013
Intercept	0.183	<.0001	0.155	0.211	1.972	<.0001	1.948	1.997
Age	0.005	<.0001	0.003	0.006	-0.004	<.0001	-0.005	-0.003
Index60	-0.283	<.0001	-0.300	-0.267	0.295	<.0001	0.281	0.310

ESM Table 1. Associations (Not Adjusted and Adjusted) of BMIp or age with HOMA-IR or Matsuda Index adjusted for Index60.

	HOMA-IR<br>N=5611				MI<br>N=5611			
Variable	Parameter Estimate	P value	95% Confidence Limits		Parameter Estimate	P value	95% Confidence Limits	
Intercept	-0.498	<.0001	-0.553	-0.443	2.606	<.0001	2.555	2.656
BMIp	0.015	<.0001	0.014	0.016	-0.014	<.0001	-0.015	-0.013
Intercept	-0.614	<.0001	-0.664	-0.564	2.706	<.0001	2.660	2.751
BMIp	0.012	<.0001	0.011	0.012	-0.011	<.0001	-0.011	-0.010
IGI	0.274	<.0001	0.259	0.289	-0.262	<.0001	-0.275	-0.248
Intercept	0.048	0.0012	0.019	0.078	2.112	<.0001	2.085	2.139
Age	0.014	<.0001	0.013	0.015	-0.014	<.0001	-0.015	-0.013
Intercept	-0.195	<.0001	-0.225	-0.166	2.337	<.0001	2.310	2.364
Age	0.010	<.0001	0.009	0.011	-0.010	<.0001	0.012	-0.009
IGI	0.284	<.0001	0.269	0.299	-0.268	<.0001	-0.281	-0.255

ESM Table 2. Associations (Not Adjusted and Adjusted) of BMIp or age with HOMA-IR or Matsuda Index adjusted IGI. Indentations of rows under Variable heading indicate adjustments of BMIp and Age for IGI.

[Linear regression models for HOMA-IR and MI]

HOMA-IR				Matsuda Index		
Not Staged to 1 (N=2399)						
Parameter	Parameter Estimate	P value	Hazard Ratio	Parameter Estimate	P value	Hazard Ratio
Tertile 2 vs.1	-0.031	0.8359	0.969	0.214	0.1753	1.239
Tertile 3 vs. 1	-0.076	0.6203	0.926	0.229	0.1446	1.258
Tertile 2 vs.1	-0.032	0.8288	0.968	0.196	0.2240	1.217
Tertile 3 vs. 1	-0.028	0.8609	0.972	0.237	0.1482	1.268
IGI (NS)	0.023	0.4543	1.024	0.035	0.3220	1.036
Stage 1 to 2 (N=3139)						
Tertile 2 vs.1	-0.131	0.1156	0.877	-0.288	0.0010	0.749
Tertile 3 vs. 1	0.148	0.0768	1.160	-0.311	0.0002	0.732
Tertile 2 vs.1	-0.064	0.4504	0.938	-0.409	<.0001	0.664
Tertile 3 vs. 1	0.285	0.0011	1.330	-0.497	<.0001	0.608
IGI (St1)	-0.124	<.0001	0.883	-0.151	<.0001	0.859

ESM Table 3. Prediction of the risk of transitioning from Not Staged to Stage 1 and from Stage 1 to 2 type 1 diabetes by tertiles of HOMA-IR and Matsuda Index after adjustment for IGI. Indentations of rows under Parameter heading indicate adjustments for Index60.

[Cox regression analysis (NT= Not Staged; St1= Stage); Not Staged (AAb<2 with normoglycemia) to Stage 1 (AAb≥2 with normoglycemia): IGI values at Not Staged used for adjustment; Stage 1 to Stage 2 (AAb≥2 associated with dysglycemia): IGI values at Stage 1 used for adjustment].

HOMA-IR				Matsuda Index		
Not Staged to 1 (N=2399)						
Parameter	Parameter Estimate	P value	Hazard Ratio	Parameter Estimate	P value	Hazard Ratio
Tertile 2 vs.1	-0.031	0.8359	0.969	0.214	0.1753	1.239
Tertile 3 vs. 1	-0.076	0.6203	0.926	0.229	0.1446	1.258
Tertile 2 vs.1	0.013	0.9305	1.013	0.186	0.2423	1.206
Tertile 3 vs. 1	0.015	0.9226	1.015	0.150	0.3498	1.162
Age (NS)	-0.019	0.0002	0.981	-0.018	0.0003	0.981
Stage 1 to 2 (N=3138)						
Tertile 2 vs.1	-0.131	0.1156	0.877	-0.288	0.0010	0.749
Tertile 3 vs. 1	0.148	0.0768	1.160	-0.311	0.0002	0.732
Tertile 2 vs.1	-0.136	0.1066	0.872	-0.276	0.0018	0.759
Tertile 3 vs. 1	0.132	0.1244	1.142	-0.292	0.0008	0.746
Age (St1)	0.001	0.6718	1.001	0.000	0.9813	1.000

ESM Table 4. Prediction of the risk of transitioning from Not Staged to Stage 1 and from Stage 1 to 2 type 1 diabetes by tertiles of HOMA-IR and Matsuda Index after adjustment for age. Indentations of rows under Parameter heading indicate adjustments for Age.

[Cox regression analysis (NT= Not Staged; St1= Stage); Not Staged (AAb<2 with normoglycemia) to Stage 1 (AAb≥2 with normoglycemia): age values at Not Staged used for adjustment; Stage 1 to Stage 2 (AAb≥2 associated with dysglycemia): age values at Stage 1 used for adjustment].

	HOMA-IR N=6114				Matsuda Index N=6256		
Parameter	Parameter Estimate	P value	Hazard Ratio	Parameter	Parameter Estimate	P value	Hazard Ratio
Tertile 2 vs.1	0.210	0.0033	1.234	Tertile 2 vs. 1	-0.418	<.0001	0.658
Tertile 3 vs. 1	0.617	<.0001	1.854	Tertile 3 vs. 1	-0.619	<.0001	0.538
IGI (baseline)	-1.466	<.0001	0.231	IGI (baseline)	-1.478	<.0001	0.228
Tertile 2 vs.1	0.354	<.0001	1.425	Tertile 1 vs. 3	-0.534	<.0001	0.586
Tertile 3 vs. 1	0.843	<.0001	2.324	Tertile 2 vs. 3	0.012	<.0001	0.402
IGI (baseline)	-1.325	<.0001	0.266	IGI (baseline)	-0.028	<.0001	0.256
Age (baseline)	-0.038	<.0001	0.962	Age (baseline)	-0.049	<.0001	0.960

ESM Table 5. Prediction of the risk of progression to Stage 3 type 1 diabetes by tertiles of HOMA-IR and Matsuda Index after adjustment for IGI and age. Indentations of rows under Parameter heading indicate adjustments of for IGI and Age.

[Cox regression analysis with baseline IGI, or IGI and age, as covariates for adjustment. Baseline: monitoring visit when the initial OGTT was performed]

SUPPLEMENTARY FIGURES

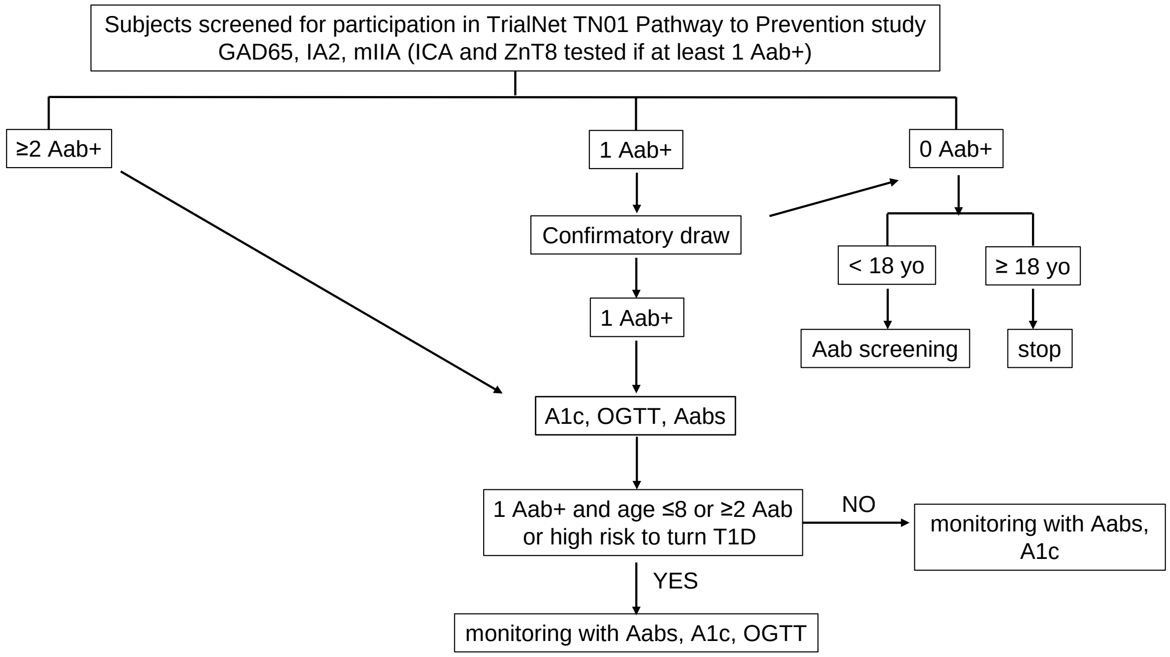

ESM Figure 1. Study design. Participants enrolled in the TN01 TrialNet Pathway to Prevention Study were first- and second-degree relatives of patients with type 1 diabetes screened for islet autoantibodies. They were tested for the presence of glutamic acid decarboxylase autoantibodies (GAD65), insulin (microinsulin antibody, mIAA) and insulinoma-associated antigen 2 (IA-2A). If tested positive for at least 1 AAb+, they were tested also for zinc transporter 8 antibody (ZnT8A) and islet cell antibody (ICA). Participants without autoantibodies (0 AAb+) younger than 18 years old were followed for autoantibody screening, whereas participants older than 18 years old were no longer monitored. Participants positive for a single autoantibody (1 AAb+, confirmed twice) or positive for 2 or more autoantibodies (≥2 AAb+), were tested for glycated haemoglobin, oral glucose tolerance test and autoantibodies. Participants with 1 AAb+ younger than 8 years old and participants with ≥2 AAb+ or at high risk to turn diabetic were monitored with autoantibodies, glycated haemoglobin and OGTT.

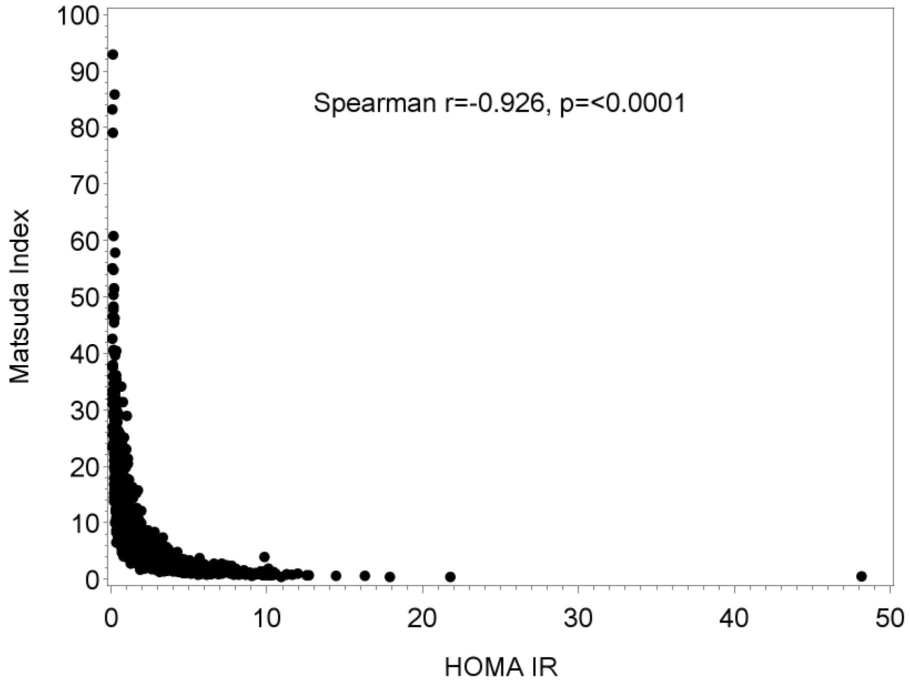

ESM Fig 2. Correlation between HOMA-IR and Matsuda Index.
Curvilinear inverse relationship is evident between HOMA-IR and Matsuda Index.
Spearman's rank correlation coefficient r=-0.926

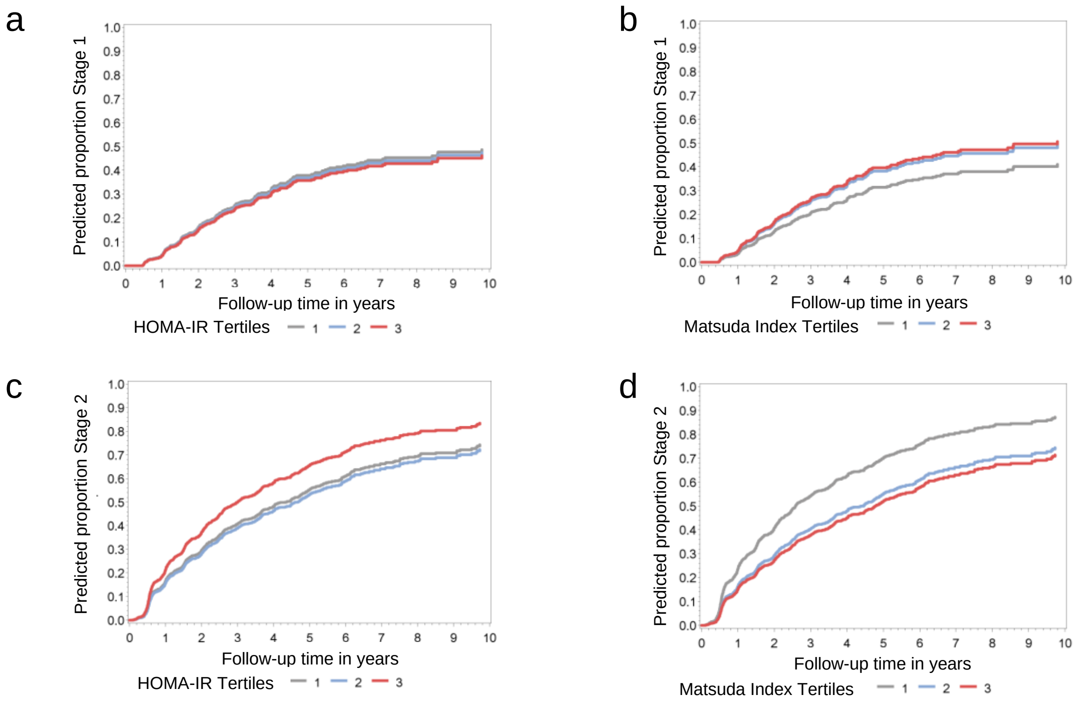

ESM Fig 3. Predicted survival functions of time from Not Staged to Stage 1 (a-b) and from Stage 1 to Stage 2 by tertiles of HOMA-IR (a-c) and Matsuda Index (b-d) based on a Cox Regression analysis adjusted for IGI. Estimates were determined based on the median values of IGI (ie. 0.76).

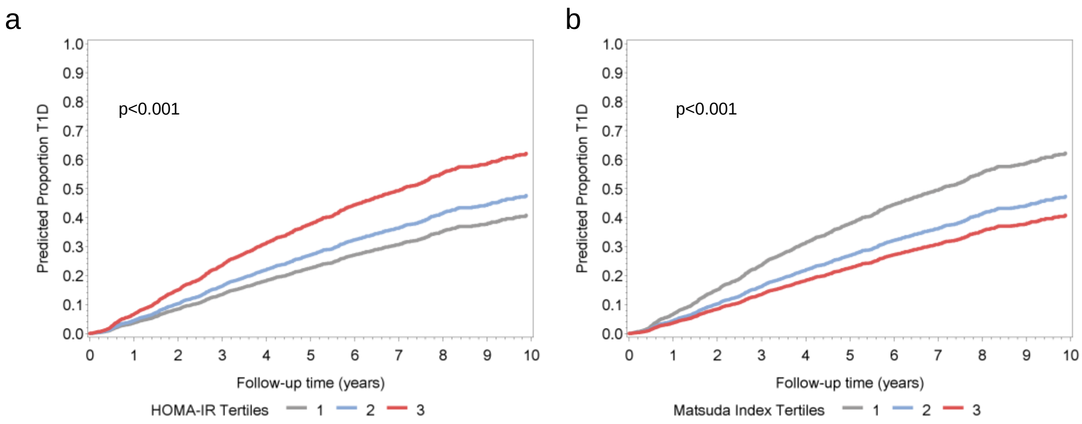

ESM Fig 4. Predicted survival functions of time to T1D by tertiles of HOMA-IR (a) and Matsuda Index (b) based on a Cox Regression analysis adjusted for IGI. Estimates were determined based on the median values of IGI (i.e. 0.76).
